# Mental health care needs of transgender people living in Russia

**DOI:** 10.1192/j.eurpsy.2022.722

**Published:** 2022-09-01

**Authors:** E. Chumakov, Y. Ashenbrenner, A. Gvozdetsky, N. Petrova, L. Azarova, O. Limankin

**Affiliations:** 1 St.-Petersburg Psychiatric Hospital No 1 named after P.P. Kaschenko, Day Inpatient Department, St Petersburg, Russian Federation; 2 Saint-Petersburg University, Department Of Psychiatry And Addictions, Saint-Petersburg, Russian Federation; 3 North-Western State Medical University named after I.I. Mechnikov, Department Of Psychiatry And Addictions, St. Petersburg, Russian Federation; 4 North-Western State Medical University named after I.I. Mechnikov, Department Of Psychotherapy, Medical Psychology And Sexology, St Petersburg, Russian Federation; 5 St-Petersburg Institute of Postgraduate Improvement of Physicians-experts of the Ministry of Labour and Social Protection, Department Of Social Psychiatry And Psychology, St Petersburg, Russian Federation

**Keywords:** Transgender, mental health care needs, gender transition, Russia

## Abstract

**Introduction:**

The majority of researchers agree that transgender people have an increased burden of mental disorders compared to the general population. However, it is strongly suggested that transgender people still do not receive mental health care that they need.

**Objectives:**

To assess the mental health care needs of transgender people living in Russia.

**Methods:**

An anonymous online survey was conducted throughout November 2019. 588 transgender adults living in all Federal Districts of Russia (mean age 24.0±6.7) were included in the final analysis.

**Results:**

It was found that 308 respondents (52.4%) had visited mental health professional prior to gender transition. 150 people (25.5%) reported to had been diagnosed with a mental disorder before gender transition, and a further 77 respondents (13.1%) indicated that they had been diagnosed with a mental disorder after transition began. 157 people (26.7%) received treatment from a mental health professional. 222 respondents (37.8%) had experience of taking medication off-prescription to improve mental wellbeing. 464 people (78.9%) reported being in need of psychological care. 289 people (49.1%) indicated that they were experiencing barriers in obtaining psychiatric (psychotherapeutic, psychological) care, which was associated with a perceived need for psychological support (OR=4.33 [95% CI: 2.49;7.80], p<0.001), being diagnosed with a mental disorder prior to gender transition (OR=2.19 [95% CI: 1.30;3.77], p=0.004), poorer housing conditions (OR=0.86 [95% CI: 0.79;0.93], p<0.001).

**Conclusions:**

Our research shows that there is a high perceived need for qualified mental health care for transgender people in Russia.

**Disclosure:**

No significant relationships.

